# Comparative Study of First Order Optimizers for Image Classification Using Convolutional Neural Networks on Histopathology Images

**DOI:** 10.3390/jimaging6090092

**Published:** 2020-09-08

**Authors:** Ibrahem Kandel, Mauro Castelli, Aleš Popovič

**Affiliations:** 1 Nova Information Management School (NOVA IMS), Campus de Campolide, Universidade Nova de Lisboa, 1070-312 Lisboa, Portugal; mcastelli@novaims.unl.pt; 2 School of Economics and Business, University of Ljubljana, Kardeljeva Ploščad 17, 1000 Ljubljana, Slovenia; ales.popovic@ef.uni-lj.si

**Keywords:** image classification, convolutional neural networks, deep learning, medical images, transfer learning, optimizers

## Abstract

The classification of histopathology images requires an experienced physician with years of experience to classify the histopathology images accurately. In this study, an algorithm was developed to assist physicians in classifying histopathology images; the algorithm receives the histopathology image as an input and produces the percentage of cancer presence. The primary classifier used in this algorithm is the convolutional neural network, which is a state-of-the-art classifier used in image classification as it can classify images without relying on the manual selection of features from each image. The main aim of this research is to improve the robustness of the classifier used by comparing six different first-order stochastic gradient-based optimizers to select the best for this particular dataset. The dataset used to train the classifier is the PatchCamelyon public dataset, which consists of 220,025 images to train the classifier; the dataset is composed of 60% positive images and 40% negative images, and 57,458 images to test its performance. The classifier was trained on 80% of the images and validated on the rest of 20% of the images; then, it was tested on the test set. The optimizers were evaluated based on their AUC of the ROC curve. The results show that the adaptative based optimizers achieved the highest results except for AdaGrad that achieved the lowest results.

## 1. Introduction

To evaluate whether tissue is cancerous, a sample is taken from the suspicious area and then evaluated, under an optical microscope, by the pathologist. This procedure is very time-consuming and extremely complicated [1], and therefore, it requires an expert pathologist with years of experience. Depending on the particular task, even an expert pathologist could make errors. This complicated procedure often demands a second opinion or even assistance, which is where artificial intelligence assumes a role. Artificial intelligence (AI) can provide significant help, whether through a lot it is automation or to furnish the pathologist with a second opinion. AI can be defined as using a computer to generate a prediction of each image by training a deep neural network model. The training process consists of feeding the system labeled pathology images, after which the algorithm seeks; first, to map a function between the input label and the prediction and second, measures the error and tries to minimize it. The state-of-the-art algorithm used in the image classification is the convolutional neural network.

In the context of image classification, deep learning may be defined as a computer program is said to learn from experience E, like pathology images, concerning some task T, like image classification that differentiates between cancerous and non-cancerous images, and is capable of recognizing the relevant image without being explicitly programmed to do so, and using a performance measure like the AUC of the ROC curve. The algorithm’s performance on the image classifier, as measured by the AUC, improves by adding more images. Practically speaking, machine learning is the task of recognizing patterns from training images and applying these patterns to identify an image with an unknown label.

The convolutional neural network (CNN) has been used as an image classification algorithm for nearly two decades [2]. The real power of CNN was rediscovered in the context of the ImageNet competition, where millions of images, with thousands of labels, were classified with 85% accuracy; at that time, CNN resumed its former role as one of the most important algorithms for image classification [3]. CNN has been applied in different image classification domains, such as agriculture [4,5,6] and traffic detection [7,8]. With the rapid improvements in GPU cards and the increasing size of datasets, many influential and robust architectures, like AlexNet [9], VGG16 [10], VGG19 [10], ResNet50 [11], and InceptionV3 [12], were introduced. Transfer learning is a deep learning technique, which allows the knowledge acquired during training on previous models to be applied to new tasks. Transfer learning has many advantages. It saves time by starting from the end point of the most recent training, instead of training the new model from scratch; it extends the knowledge it acquired from previous models; transfer learning is particularly useful when the size of the new training dataset is small. Transfer learning has made significant contributions to the fields of computer vision, audio classification, and natural language processing.

The difference between the predicted label and the correct label is called the cost function; the whole point of the algorithm is to minimize this cost function. As the algorithm most commonly used to minimize the cost function, backpropagation is an iterative algorithm, where each of its iterations consists of two passes: A forward pass throughout the entire network, where the inputs are propagated from the input layer to the output layer. At this point, the cost function is be calculated to measure the performance of the network; then there is the backward pass, where the weights are backpropagated from the output to the input of the network. The optimizers are used to minimize this cost function.

This work evaluates different first-degree optimizers used to classify pathology images as cancerous or non-cancerous. Each optimizer is evaluated based on its performance and convergence time. Four CNN architectures will be used to compare the performance of each optimizer to those of the others.

## 2. Related Works

Many works compared the performance of different optimizers in the context of different neural network architectures; the reported approaches differ in relation to the network architecture, datasets, and the optimizers under study.

In a study by Dogo et al. [13], the authors evaluated the performance of seven optimizers on three image datasets: Natural Images dataset, Cats and Dogs dataset, and Fashion MNIST dataset. The authors evaluated the performance of each optimizer based on accuracy achieved and the convergence time, where convergence consists of reaching the minimum of the function. To determine the performance quality of each optimizer, the authors proposed a simple CNN architecture, with three convolutional layers, and one dense layer with 64 neurons. For the Cats and Dogs dataset, the Nadam optimizer achieved the best performance, and the Adadelta optimizer produced the most mediocre performance; the RMSProp represents the shortest convergence time, and the Nadam optimizer achieved the longest convergence. For the Fashion dataset, the Adam optimizer achieved the highest degree of accuracy, and the Adadelta optimizer displayed the lowest accuracy; the Adamax optimizer achieved the shortest convergence time, and the Adadelta optimizer had the longest convergence time was the Adadelta optimizer. For the Natural dataset, the Nadam optimizer was the best performer, and the Adagrad optimizer exhibited the most inferior accuracy; the SGD algorithm achieved the shortest convergence time, and the Adadelta algorithm had the longest convergence time. The authors concluded that the Nadam optimizer was the best of all tested optimizer, due to its combined mastery of the momentum and the adaptive gradient estimation.

The authors Prilianti et al. [14] compared the performance of seven optimizers on the digital plant dataset. To evaluate each optimizer, the authors used three CNN architectures; the first was a shallow network with only one convolutional layer and without any dense layers; the second CNN architecture used was the LeNet architecture, which was introduced by Lecun et al., [15]; and the third CNN architecture was the AlexNet [9]. The authors evaluated the performance of each optimizer on each CNN architecture, based on the mean square error (MSE). The Adam optimizer achieved the lowest MSE for the shallow net architecture, as well as the LeNet architecture, while the Adadelta achieved the lowest MSE on the AlexNet architecture. The authors concluded that Adam optimizer achieved the best performance.

Jangid and Srivastava [16] assessed the performance of three optimizers on handwritten Devanagari characters. The optimizers tested were Adam, Adamax, and RMSProp. To evaluate each optimizer, the authors introduced a CNN architecture with three convolutional layers and one dense layer with 1000 neurons. For this architecture, RMSProp achieved the best accuracy. Swastika et al. [17] evaluated three optimizers to classify vehicle types: Adam, Adadelta, and SGD. The authors used three CNN architectures to evaluate each optimizer: a shallow network, LeNet, and MiniVGGNet. The optimizers were evaluated based on their accuracy, which meant that the Adadelta optimizer was the best for the Mini VGGNet architecture.

This study uses four CNN architectures to perform a comparative evaluation of six first-degree stochastic gradient descent optimizers: the optimizers tested are Nesterov gradient descent, Adagrad, Adam, Adamax, Nadam, and RMSProp; and the CNN architectures tested are VGG16, InceptionV3, DenseNet, and ResNet50. The optimizers are evaluated based on their AUC of the ROC curve and their convergence time. All the optimizers’ default hyperparameters were kept constant throughout the experiment, except the learning rate, which was set to three values 0.001, 0.0001, and 0.0001. Fine-tuning was applied to each network to adjust its weight to the new dataset.

## 3. Methodology

### 3.1. Dataset

**Figure 1:**
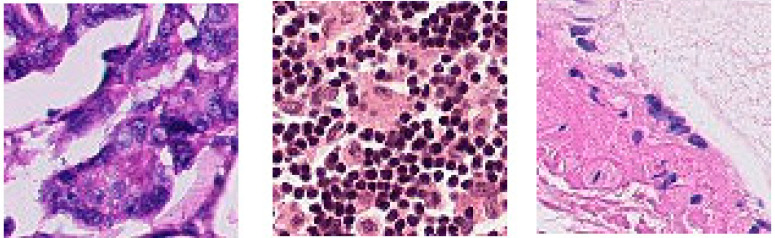
Example of images available in the PatchCamelyon dataset [20].

The public available PatchCamelyon dataset [18,19] was used in this study. The images represent sentinel axillary lymph nodes to investigate the spread of breast cancer. The dataset was sampled from two hospitals in the Netherlands, experienced pathologists from the Netherlands annotated the dataset labels. The dataset was acquired from the Kaggle platform [20]. The dataset consists of 220,025 images to train the classifier; the dataset is composed of 60% positive images and 40% negative images, and it includes 57,458 unlabeled images to test the classifier performance. All images have dimensions of 96 × 96 pixels. Eighty percent of the dataset is used to train the classifier, which is subsequently evaluated with the other 20% of the dataset images; the classifier is also tested on the online set of 57,458 images, and the results are uploaded to the Kaggle platform to detect the model performance. A sample of images is presented in Figure 1.

### 3.2. Convolutional Neural Networks

CNN is the most used algorithm in image classification, where it is understood to be a deep learning algorithm that serves as a feed-forward neural network with more than one hidden layer. The CNN for image classification was introduced by Fukushima [2] to mimic the biological visual cortex of the brain. CNN combines sophisticated features obtained from the higher layers of the network with the generic features obtained from the lower layers of the network. The most critical layer of CNN is the convolution layer, which is responsible for capturing the temporal and spatial information of each image; the convolutional layer must conduct the convolution operation, which is a mathematical operation performed between the input and the filter to produce the feature map. Equation (1) shows the convolution operation,

(1) $$O\left[u,v\right]=F\left[m,n\right]*I\left[u,v\right]=\sum_{m}^{\;}\sum_{n}^{\;}F\left[m,n\right]\;\cdot I\left[u+m,v+n\right]\;$$

where F[m,n] is the convolution filter, I[u,v] is the input image and O[u,v] is the output feature map.

A filter is convolved over the input image to produce a feature map. Another CNN layer is the activation function, which is used to present non-linearity because usually, the image classification task is highly non-linear. To reduce overfitting and to reduce the spatial footprint of each filter, two main techniques can be used to extract the essential pixels and removing the noise. The first involves using a stride value larger than 1, which reduces the output of each filter. The second technique is called pooling, where a pooling layer usually follows the activation layer. Pooling layers can strengthen network spatial invariance [21]. The two main types of pooling layers are the maximum pooling layer and the average pooling layer.

Then, fully connected layers follow, usually defined at the end of the network, which takes the output of the feature extraction layers. The primary purpose of the dense layer is to consider all the features extracted from the previous layers and employ these features to classify the output. The dense layers are followed by an activation function, which usually consists of a rectified linear unit (ReLU) layer; finally, at the end of the network, a softmax or sigmoid function is used to output the target probability.

### 3.3. Optimizers

The model learns (trains) on a given dataset by comparing the actual label of the input (available in the training set) to the predicted label, thereby, minimizing the cost function. Hypothetically, if the cost function is zero, the model has learned the dataset correctly. However, an optimization algorithm is needed to achieve the minimum of a cost function. The next section discusses different optimization algorithms, introduced in the literature, to minimize the cost function.

#### 3.3.1. Vanilla Gradient Descent Optimizers

Gradient descent is the primary class of optimizers capable of finding the minimum value of the cost function. The literature has introduced three versions of gradient descent.

##### Batch Gradient Descent

The first optimization algorithm was the batch gradient descent optimization algorithm (BGD), which updates the network weights after scanning the whole training dataset; in the case of images, convergence takes much time, as there may be millions of weights to optimize and the whole dataset needs to be reevaluated at every step (i.e., epoch). For convex loss function, it is guaranteed that the BGD will converge to the global minimum, while it converges to a local minimum for non-convex functions. The weights are updated based on Equation (2):

(2) $$w_{t+1}=w_{t}-\eta \frac{\partial C}{\partial w_{t}}\;$$

(3) $$\frac{\partial C}{\partial w_{t}}=\;\nabla_{w}\;C\left(w_{t}\right)\;$$

where Equation (3) is the gradients update equation, and η is the learning rate hyperparameter. $w_{t}$ are the weights at step t, C(.) is the cost function and $\nabla_{w}\;C\left(w_{t}\right)$ is the gradient of weight parameters $w_{t}$.

##### Stochastic Gradient Descent

To overcome the shortcomings of BGD, stochastic gradient descent (SGD) was introduced. SGD allows to update the network weights per each training image, that is why SGD is sometimes called online training. However, such updates engender massive fluctuation in the loss function, due to the high variance between different images, which can create much noise in the training phase:

(4) $$w_{t+1}=w_{t}-\eta \frac{\partial C}{\partial w_{t}}\;$$

(5) $$\frac{\partial C}{\partial w_{t}}=\;\nabla_{w}\;C\left(w_{t};x^{\left(i\right)};y^{\left(i\right)}\right)\;$$

The weights are updated based on Equation (4), where Equation (5) is the gradient update equation, and η is the learning rate hyperparameter. $w_{t}$ are the weights at step t, C(.) is the cost function, and $\nabla_{w}\;C\left(w_{t}\right)$ is the gradient of weight parameters $w_{t}$ for image x and its corresponding label y.

##### Mini-Batch Gradient Descent

Mini-batch gradient descent was introduced to overcome the shortcomings of the previous two algorithms, because it allows for the weights to be updated per batch, and not per image. As such, mini-batch gradient descent may be regarded as a particular case of SGD, where the number of samples is more than one. In the literature and it follows, in this paper, the mini-batch is referred to as stochastic gradient descent (SGD):

(6) $$w_{t+1}=w_{t}-\eta \frac{\partial C}{\partial w_{t}}\;$$

(7) $$\frac{\partial C}{\partial w_{t}}=\;\nabla_{w}\;C\left(w_{t};x^{\left(i:i+n\right)};y^{\left(i:i+n\right)}\right)\;$$

The weights are updated based on Equation (6), and Equation (7) is the gradient update equation. η is the learning rate hyperparameter, $w_{t}$ are the weights at step t, n is the number of data points, C(.) is the cost function and $\nabla_{w}\;C\left(w_{t}\right)$ is the gradient of weight parameters $w_{t}$ for image x and its corresponding label y.

#### 3.3.2. Momentum-Based Gradient Descent Optimizers

The main drawback of using mini-batch SGD is the presence of oscillations during the updating of the weights. These oscillations usually result in a long time to reach convergence. Momentum, also known as moving average gradients, was introduced, in order to overcome this issue and to fix the gradients’ direction.

##### Momentum Gradient Descent

Understanding the right direction for the gradient avoids oscillations in the wrong directions, and knowing the right direction relies on using the previous position for guidance. Considering the previous position, the updating rule adds a fraction of the previous update, which gives the optimizer the momentum needed to continue moving in the right direction. The weights are updated based on Equation (10),

(8) $$V_{t}=\lambda V_{t-1}+\eta \frac{\partial C}{\partial w_{t}}\;$$

(9) $$\frac{\partial C}{\partial w_{t}}=\;\nabla_{w}\;C\left(w_{t};x^{\left(i:i+n\right)};y^{\left(i:i+n\right)}\right)\;$$

(10) $$w_{t+1}=w_{t}-V_{t}\;$$

where V is the velocity, and it is initialized to 0. λ is used to select the amount of information needed from the previous update. η is the learning rate hyperparameter, $w_{t}$ are the weights at step t, n is the number of data points, C(.) is the cost function, and $\nabla_{w}\;C\left(w_{t}\right)$ is the gradient of weight parameters $w_{t}$ for image x and its corresponding label y.

##### Nesterov Momentum Gradient Descent

If the momentum is sufficiently high, close to the minimum, the optimizer may overshoot the minimum. The previous optimization algorithms take the current and the previous gradients into account for updating the weights. However, to make the optimization algorithm more robust, we must take the future gradients into account as well, to approximate the gradients’ direction. The weights are updated based on Equation (13),

(11) $$V_{t}=\lambda V_{t-1}+\eta \frac{\partial C}{\partial w_{t}}\;$$

(12) $$\frac{\partial C}{\partial w_{t}}=\;\nabla_{w}\;C\left(w_{t}-\lambda V_{t-1};x^{\left(i:i+n\right)};y^{\left(i:i+n\right)}\right)\;$$

(13) $$w_{t+1}=w_{t}-V_{t}\;$$

where V is the velocity, and it is initialized to 0, λ is used to select the amount of information needed from the previous update. While, η is the learning rate hyperparameter, $w_{t}$ are the weights at step t, n is the number of data points, C(.) is the cost function, and $\nabla_{w}\;C\left(w_{t}\right)$ is the gradient of weight parameters $w_{t}$ for image x and its corresponding label y. $\left(w_{t}-\lambda V_{t-1}\right)$ is the look-ahead position that is capable of approximating the next gradient position, thereby allowing it to slow down if it threatens to overshoot the minimum.

#### 3.3.3. Adaptive Gradient Descent Optimizers

All the optimization mentioned above has a fixed learning rate, while, in practice, deep learning algorithms are non-convex problems. That may be a problem, as we may face a sparse weight matrix, where we require different updates for different weights, especially for infrequent weights, where significant updates are needed to reach to avoid oscillating.

##### AdaGrad Optimizer

To scale the learning rate for each weight, the AdaGrad optimization algorithm [22] was introduced to establish different updates for different weights. The learning rate is tuned automatically, by dividing the learning rate by the sum of squares of all previous gradients. The weights are updated based on Equation (14),

(14) $$w_{t}^{i}=w_{t-1}^{i}-\frac{\eta }{\sqrt{\sum_{T=1}^{t}{\left(\nabla_{w}C\left(w_{T}^{i}\right)\right)}^{2}+\epsilon }}\cdot \nabla_{w}C\left(w_{t}^{i}\right)\;$$

where  η is the learning rate hyperparameter, $w_{t}$ are the weights at step t, C(.) is the cost function, and $\nabla_{w}\;C\left(w_{t}\right)$ is the gradient of weight parameters $w_{t}$ for image x and its corresponding label y. The sum of squares $\sqrt{\sum_{T=1}^{t}{\left(\nabla_{w}C\left(w_{T}^{i}\right)\right)}^{2}}$ is used to scale the learning rate; it gives a high learning rate for the least frequent gradients and a low learning rate for the more frequent gradients.

##### RMSProp Optimizer

The main drawback of AdaGrad is that the learning rate decreases monotonically because every added term is positive. After many epochs, the learning rate is so small that it stops updating the weights. RMSProp was introduced to address the problem of the monotonically decreasing learning rate [23]. The weights are updated based on Equation (17),

(15) $$G=\nabla_{w}C\left(w_{t}\right)\;$$

(16) $$E{\left[G^{2}\right]}_{t}=\lambda \;E{\left[G^{2}\right]}_{t-1}+\left(1-\lambda \right)G_{t}^{2}\;$$

(17) $$w_{t}^{i}=w_{t-1}^{i}-\frac{\eta }{\sqrt{E{\left[G^{2}\right]}_{t}}+\epsilon }\cdot \nabla_{w}C\left(w_{t}^{i}\right)\;$$

where  η is the learning rate hyperparameter, $w_{t}$ are the weights at step t, C(.) is the cost function, and $\nabla_{w}\;C\left(w_{t}\right)$ is the gradient of weight parameters $w_{t}$ for image x and its corresponding label y. λ is used to select the amount of information needed from the previous update. $E{\left[G^{2}\right]}_{t}\;$ is the running average of the squared gradients, which has been used to avoid the monotonically decreasing gradients of the AdaGrad optimizer.

##### Adam Optimizer

The Adam optimization [24] algorithm was introduced to combine the benefits of Nesterov momentum, AdaGrad, and RMSProp algorithms. The weights are updated based on Equation (18):

(18) $$w_{t}^{i}=w_{t-1}^{i}-\frac{\eta }{\sqrt{{\hat{v}}_{t}}+\epsilon }\cdot {\hat{m}}_{t}\;$$

where:

(19) $${\hat{m}}_{t}=\frac{m_{t}}{1-\beta_{1}^{t}}\;$$

(20) $${\hat{v}}_{t}=\frac{v_{t}}{1-\beta_{2}^{t}}\;$$

(21) $$m_{t}=\beta_{1}m_{t-1}+\left(1-\beta_{1}\right)G\;$$

(22) $$v_{t}=\beta_{2}v_{t-1}+\left(1-\beta_{2}\right){\left[G\right]}^{2}\;$$

(23) $$G=\nabla_{w}C\left(w_{t}\right)\;$$

where  η is the learning rate hyperparameter, $w_{t}$ are the weights at step t, C(.) is the cost function, and $\nabla_{w}\;C\left(w_{t}\right)$ is the gradient of weight parameters $w_{t}$ for image x and its corresponding label y, $\beta_{i}$ is used to select the amount of information needed from the previous update, where $\beta_{i}\in \left[0,1\right]$, $m_{t}$ is the running average of the gradients, also known as the first moment, $v_{t}$ is the running average of the squared gradients, and known as the second moment. If the first and second moments get initialized at zero, they are biased toward it, to solve this zero-biased problem, these moments are bias-corrected by dividing them by their respective β.

##### Adamax Optimizer

Adamax [24] is the update of the Adam algorithm, where the uncentered variance tends to ∞. The weights are updated based on Equation (24):

(24) $$w_{t}^{i}=w_{t-1}^{i}-\frac{\eta }{v_{t}+\epsilon }\cdot {\hat{m}}_{t}\;$$

where:

(25) $${\hat{m}}_{t}=\frac{m_{t}}{1-\beta_{1}^{t}}\;$$

(26) $$v_{t}=\max\left(\beta_{2}\cdot v_{t-1},\left|G_{t}\right|\right)\;$$

(27) $$m_{t}=\beta_{1}m_{t-1}+\left(1-\beta_{1}\right)G\;$$

(28) $$G=\nabla_{w}C\left(w_{t}\right)\;$$

where  η is the learning rate hyperparameter, $w_{t}$ are the weights at step t, C(.) is the cost function, and $\nabla_{w}\;C\left(w_{t}\right)$ is the gradient of weight parameters $w_{t}$ for image x and its corresponding label y. $\beta_{i}$ is used to select the amount of information needed from the previous update, where $\beta_{i}\in \left[0,1\right]$. $m_{t}$ is the first moment, $v_{t}$ is the second moment.

##### Nadam Optimizer

Nadam [25] is an extension of the Adam algorithm by combining it with Nesterov momentum gradient descent. The weights are updated based on Equation (29):

(29) $$w_{t}^{i}=w_{t-1}^{i}-\frac{\eta }{\sqrt{v_{t}+\epsilon }}\cdot {\tilde{m}}_{t}\;$$

where:

(30) $${\tilde{m}}_{t}=\;\beta_{1}^{t+1}{\hat{m}}_{t}+\left(1-\beta_{1}^{t}\right){\hat{g}}_{t}\;$$

(31) $${\hat{m}}_{t}=\frac{m_{t}}{1-\prod_{i=1}^{t}\beta_{1}^{i}}\;$$

(32) $${\hat{g}}_{t}=\frac{g_{t}}{1-\prod_{i=1}^{t}\beta_{1}^{i}}\;$$

where  η is the learning rate hyperparameter, and $w_{t}$ are the weights at step t. While, $\beta_{i}$ is used to select the amount of information needed from the previous update, where $\beta_{i}\in \left[0,1\right]$, $m_{t}$ is the first moment.

##### Fine-Tuning

According to [26,27] transfer learning can be formalized as follows: by having a domain *D*, where D={X,P(X)}, in which X is the feature space and P(X) is the marginal probability distribution and task T={Y,f(.)}, where Y is the label space and f(.) is the predictive function which models  P(y|x) for y ∈Y and x ∈X. By having a source domain $D_{s}\;$ and learning task $T_{S}$ and a target domain $D_{T}$ and learning task $T_{T}$, by using weights from $D_{S}\;$ and $T_{S}$, learning the target predictive function f(.) in $T_{T}$ can be improved a lot, where $D_{S}\neq D_{T}$, $T_{S}\neq \;T_{T}$, or both. Fine-tuning is very important in the classification of medical images, because the neural network usually needs many images to be trained. However, in the medical field, for many reasons, labeled medical images are scarce. Instead of initializing the weights from scratch, ImageNet weights can be used. In this paper, the networks were trained on ImageNet dataset and all the networks blocks were fine-tuned using the PatchCamelyon dataset [18,19].

#### 3.3.4. VGG16 Network

VGG16 [10] was introduced in 2014 by the researchers at Oxford’s Visual Geometry Group. It was one of the top algorithms involved in the ImageNet classification challenge, and it had an 8.1% error rate. VGG16 consists of five convolution blocks, where the first block contains two convolution layers, stacked together with 64 filters. The second block consists of two stacked convolution layers with 128 filters, where the second convolution block is separated from the first block by a max pool layer. The third block consists of three convolution layers, stacked together with 256 filters and separated from the second block by another max pool layer. The fourth and fifth layers have the same architecture, but instead, have 512 filters. The convolution filter used throughout this network is of size 3 × 3 and stride of 1. Then, a flatten layer is added between the convolution blocks and the dense layers, converting the 3D vector into a 1D vector. The last block consists of three dense layers, each of which has 4096 neurons, to classify each image. The last layer is a softmax layer, which is used to ensure that the probability summation of the output is one. ReLU was used as an activation layer throughout the network. This network was trained on the ImageNet dataset for three weeks on four GPUs to detect the ImageNet classification task. A summary of VGG16 network is presented in Table 1. 

#### 3.3.5. InceptionV3 Network

**Figure 2:**
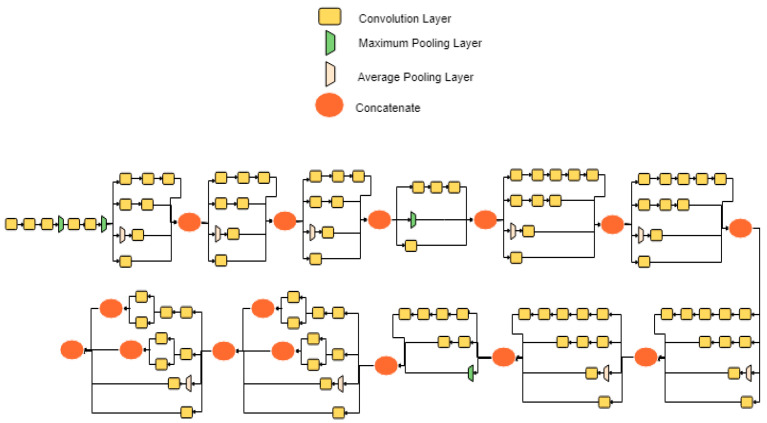
InceptionV3 network architecture.

The authors Szegedy et al. [28] introduced a novel architecture, called Inception, to participate in the ImageNet competition in 2015; Inception had an accuracy rate of 92.2%. The architecture consists of 48 layers and total parameters of 22,000,000. This architecture has a concatenated layer of convolutions, stacked in parallel to decrease the size of the architecture while maintaining its complexity. InceptionV3 network architecture is shown in Figure 2. A summary of InceptionV3 network is presented in Table 1.

#### 3.3.6. ResNet Network

The authors, He et al. [11], investigated the effect of increasing the depth of the convolutional neural network and its impact on network performance. The authors noticed that increasing the depth of the network decreases the generalizability of the network, which means that the test error of the network is higher than a shallow network. This may be due to the vanishing gradients, where the weights are not updated in deep layers. Therefore, He et al. [11] introduced a novel architecture called ResNet, where Res signifies the application of a residual connection between the convolutional layers, which is then passed to the ReLU activation layer. One of the main benefits of adding the residual connection is that the weights learned from the previous layers can be carried to the next layers during the backpropagation step. ResNet won the ImageNet competition in 2015 with Top-5 accuracy of 94.29%. It has a total of 23,587,712 parameters, and its ImageNet weights are available in the Keras package. A summary of ResNet network is presented in Table 1.

#### 3.3.7. DenseNet Network

DenseNet network [29] was inspired by the residual connection of the ResNet architecture. All the layers are connected to all their subsequent layers, meaning that a residual connection is established between all the layers. Merging will be used instead of adding to combine the layers. DenseNet has many variants depends on the number of layers; some of the variants are DenseNet 121, DenseNet169, and DenseNet201. In this paper, we opted for the DenseNet121 network. A summary of DenseNet network is presented in Table 1.

### 3.4. Overcoming Overfitting

Overfitting generally consists of memorization of the training dataset and usually leads to poor performance on the test dataset. This means that the performance on the training set can be excellent, but the performance on the test set is quite poor. The loss of the generalizability of the network may be due to many issues, such as the capacity of the network or the nature of the training dataset itself. Many measures have been introduced in the literature to overcome overfitting. Below are some techniques that were used in this research to overcome overfitting.

#### 3.4.1. Dropout

A regularization layer introduced by [30] can be applied to any layer in the network. During network training, some neurons are disabled with a pre-defined dropout-rate probability P. This can be understood as a sort of bagging for neural networks.

#### 3.4.2. Image Augmentation

Increasing the size of the training set improves the performance of the network. For image datasets, many duplicates can be created with simple changes to the original dataset, including rotation, flipping, zooming, and cropping. These transformations make the network more robust in defending against overfitting, and it enhances network performance as well. In our case, the original images are flipped, rotated, zoomed, and shifted. The rotation range used was 180°; and the images were randomly flipped horizontally and vertically; the shifting range used was 25%; and the zoom range used was 40%.

#### 3.4.3. Early Stopping

Early stopping is a precautionary measure used to prevent the network from overfitting, which may be defined as stopping the training phase of the network when the performance on the validation set stops improving for a pre-defined number of epochs. This pre-defined number usually ranges from 10–50 epochs. In our case, the number of epochs is 10.

### 3.5. Evaluation Metrics

To assess the quality of the trained CNN, many measures have been developed. For classification tasks, a confusion matrix is constructed to assess the model quality; it categorizes the model predictions, according to whether they match the correct label of the image. It has four central values:

TP: True positive (A positive example, classified as a positive example)
TN: True negative (A negative example, classified as a negative example)
FP: False positive (A negative example, but classified as a positive example)
FN: False-negative (A positive example, but classified as a negative example)

To visualize the model performance, the ROC curve was introduced to examine the trade-off between sensitivity and specificity visually. The main idea of the ROC curve is to plot the specificity of the algorithms, which is the percentage of the correctly classified negatives against the sensitivity, which is the percentage of the correctly classified positives of the algorithm [31]. The ROC curve has a diagonal line, which represents a random guess model. It means that the model cannot differentiate between true positives and false positives; this diagonal line can be considered as the baseline where models can be judged. The best model has a curve that passes through the top left corner for “100% Sensitivity” and has a 0% false-positive rate. To measure the quality of the model using the ROC curve, a statistic known as AUC or “Area under the ROC curve,” is used; this treats the ROC diagram as a two-dimensional square and measures the area under the curve. AUC has a minimum value of 0.5 and a maximum value of 1, where 0.5 represents a model with no predictive power, and 1 represents a model with 100% predictive power. According to Vuk [32], the AUC is calculated by Equation (33):

(33) $$\mathrm{AUC}=\int_{0}^{1}\frac{\mathrm{TP}}{P}\;d\frac{\mathrm{FP}}{N}=\frac{1}{\mathrm{PN}}\;\int_{0}^{N}\mathrm{TP}\;\mathrm{dFP}\;$$

where TP+FN=P and TN+FP=N.

## 4. Results

The following section details the results obtained from training the four network architectures using the six selected optimizers with three learning rates, namely, $1\times 10^{-3}$, $1\times 10^{-4}$, and $1\times 10^{-5}$. Many experiments have been conducted on this dataset, to determine the behavior of each optimizer with each network architecture to determine the best combination. The performance of each optimizer with the VGG16 architecture is presented in Table 2, with the InceptionV3 architecture in Table 3, with the ResNet architecture in Table 4, and with the DenseNet architecture in Table 5. To test the performance of each configuration, two types of evaluation were used; the first consists of splitting the training dataset into 80%/20% to train and validate the dataset. After training, the model is used to predict the class of the images in the test set, and the result is submitted to the Kaggle platform to assess the performance of each model. The AUC of the ROC curve measured the performance. The optimizers are ranked based on their test AUC that was acquired from the Kaggle platform.

In all the experiments, the default settings of each optimizer were chosen, except the learning rate, and image augmentation used for rotating, flipping, and cropping all the images. The size of the images was kept constant at 96 × 96; a batch size of 64 images was used, and early stopping was applied with the number of epochs equal to 10.

### 4.1. VGG16 Architecture Result

Table 2 shows the results for the VGG16 architecture, which shows that the highest AUC was achieved by Adam optimizer, which also took the shortest time to achieve convergence. At the same time, the lowest test AUC was achieved by RMSProp and Adamax optimizers that did not converge at all. For the highest learning rate ($1\times 10^{-3}$) the highest AUC achieved was by the Adam optimizer, and the lowest AUC achieved was by the RMSProp and Adamax optimizers. For the medium learning rate ($1\times 10^{-4}$) the highest AUC achieved was by NAG optimizer, and the lowest AUC achieved was by both AdaGrad and Adam optimizers. For the lowest learning rate ($1\times 10^{-5}$ The AdaGrad optimizer achieved), the highest AUC achieved by the Adam optimizer and the lowest AUC. Overall, the medium learning rate achieved the best results, followed by the lowest learning rate. Adam optimizer was the most stable optimizer with high results and low variance between different learning rates.

### 4.2. InceptionV3 Architecture Result

Table 3 shows the results for the InceptionV3 architecture, which shows that the highest AUC was achieved by the RMSProp optimizer, which also took the shortest time to achieve convergence. At the same time, the lowest test AUC was achieved by AdaGrad optimizer, which also took the longest time to convergence. For the highest learning rate ($1\times 10^{-3}$), the AdaGrad optimizer achieved the highest AUC, while, the Adam optimizer achieved the lowest AUC. For the medium learning rate ($1\times 10^{-4}$) the highest AUC achieved was by the RMSProp optimizer, and the lowest AUC achieved was by the AdaGrad optimizer. For the lowest learning rate ($1\times 10^{-5}$ The AdaGrad optimizer achieved), the highest AUC achieved by the AdaMax optimizer and the lowest AUC. Overall, the medium learning rate achieved the best results, followed by the lowest learning rate. Adamax optimizer was the most stable optimizer with high results and low variance between different learning rates.

### 4.3. ResNet Architecture Result

Table 4 shows the results for the ResNet architecture, which shows that the best AUC was achieved by the Nadam optimizer, while the AdaGrad optimizer achieved the lowest AUC. For the highest learning rate ($1\times 10^{-3}$) the highest AUC achieved was by the AdaGrad optimizer, and the lowest AUC achieved was by the RMSProp optimizer. For the medium learning rate ($1\times 10^{-4}$) the highest AUC achieved was by the NAG optimizer, and the lowest AUC achieved was by the AdaGrad optimizer. For the lowest learning rate ($1\times 10^{-5}$ The AdaGrad optimizer achieved), the highest AUC achieved by the Nadam optimizer and the lowest AUC. Overall, the medium learning rate achieved the best results, followed by the lowest learning rate. Adamax optimizer was the most stable optimizer with high results and low variance between different learning rates.

### 4.4. DenseNet Architecture Result

Table 5 shows the results for the DenseNet architecture, where the best AUC was achieved by Adamax optimizer, while the Adam optimizer achieved the lowest AUC. For the highest learning rate ($1\times 10^{-3}$) the highest AUC achieved was by the AdaGrad optimizer, and the lowest AUC achieved was by the Adam optimizer. For the medium learning rate ($1\times 10^{-4}$) the highest AUC achieved was by Adamax optimizer, and the lowest AUC achieved was by the Adam optimizer. For the lowest learning rate ($1\times 10^{-5}$ The AdaGrad optimizer achieved), the highest AUC was achieved by the RMSProp optimizer and the lowest AUC. Overall, the medium learning rate achieved the best results, followed by the lowest learning rate. Adamax optimizer was the most stable optimizer with high results and low variance between different learning rates.

Overall, in terms of performance across all four networks, the highest results were achieved by the adaptive learning optimizers, like Adam, Adamax, Nadam, and RMSProp. However, these optimizers needed a lower learning rate to be able to converge, while the high learning rate did not achieve good results. One exception was the AdaGrad optimizer that did not achieve high results with a low learning rate; on the contrary, it needed a high learning rate to be able to converge to an acceptable result. From our results and the results obtained by Prilianti et al. [14], it is apparent that every combination of network and optimizer will produce a unique combination. However, the general behavior of each optimizer can be noted, which can be concluded from the results. Overall, NAG optimizer did achieve high results overall the four architectures and overall the three learning rates used with the medium learning rate $\left(1\times 10^{-4}\right)$ achieved the best results. The AdaGrad optimizer did not achieve high results compared to other optimizers used, especially when trained using low learning rates. RMSProp optimizer did achieve high results with a low learning rate but was unstable with high learning rates. Adam optimizer needed a low learning rate to be able to converge to high results. While, the AdaMax optimizer behaved similarly to the Adam optimizer, except for a high learning rate with the VGG16, where it did not converge at all, one reason may be the shallow depth of the VGG16 network. Nadam optimizer did achieve high results with both the medium learning rate and the low learning rate.

## 5. Discussion

Taking into account the results achieved from the experimental campaign, it is possible to draw some interesting observations of the behavior of the CNNs and optimizers considered in this paper. By focusing on the choice of the optimizer and its relation with the learning rate, the experimental results confirm that the choice of the learning rate may result in an unstable behavior of the training process. This is particularly evident, for some of the considered networks and optimizers, when considering the smallest learning rate used in the experiments. As one can see, when $\;\mathrm{LR}=10^{-3}$, the training process of VGG16 with both RMSProp and Adamax optimizer result in a poor performance of the model. As explained in the previous sections, this can be motivated by the fact that the weights of the network change abruptly from one epoch to the next. Moving to lower LR values allows the convergence of the training process in all the configurations that were investigated. Overall, the results match the theoretical expectation: A lower LR value allows for a smoother convergence, but it requires more time with respect to a greater LR value.

Another interesting observation relates to the importance of the hyperparameters. While this is a topic of fundamental importance in the area of deep learning, it is particularly evident from the results of the experimental phase. In particular, all the considered architecture produced a comparable performance when the best configuration of the learning rate and optimizer (that is different for each type of architecture) was considered. In other words, it seems that the choice of the hyperparameters not only plays an essential role in determining the performance of the model, but the CNNs under exam are indistinguishable in terms of performance. We believe that this is an interesting observation that should further stress the importance of the tuning of the hyperparameters.

Focusing on the optimizers, AdaGrad produces the best performance with $\mathrm{LR}=10^{-3}$ and, under this aspect, it behaves differently with respect to the other optimizers under analysis. Conversely, Adam, Adamax, and Nadam obtained the best performance on the considered CNNs when $\mathrm{LR}=10^{-5}$ (except Adamax on the DenseNet architecture, where the best performance is obtained with $\mathrm{LR}=10^{-4}$).

Globally, the best result on the considered dataset was achieved by Adamax optimizer and DenseNet network. Anyway, the differences in terms of performance among the best configurations of each network, are not statistically significant. Overall, every optimizer behaved differently according to the particular architecture. For instance, for the deep architectures like ResNet, AdaGrad outperformed Adam and Adamax. For the shallow architectures like VGG16, AdaMax and NAG had the same performance. Given a specific network, each optimizer requires a different amount of time for converging (i.e., concluding ten epochs). In particular, RMSProp was the fastest optimizer, whereas training a CNN with AdaGrad resulted in the slowest training process. This result is coherent with respect to the one discussed proposed by Dogo et al. [13], in which the authors investigated the effect of different optimizers in terms of required time to reach. More in detail, training the VGG16 architecture requires a minimum of 90 min (RMSProp optimizer and learning rate of 10^−3^) and a maximum of nine hours (AdaGrad optimizer and learning rate of 10^−5^). InceptionV3 requires approximately one hour more than VGG16; in this case, the use of RMSProp (with a learning rate of 10^−3^) resulted in the fastest training process (approximately 150 min), while the use of AdaGrad (with a learning rate of 10^−5^) required approximately 10 h to finish. An identical pattern was observed for ResNet and DenseNet, that are requiring approximately two hours more than VGG16 for concluding the training process.

Finally, it is important to compare the results achieved with transfer learning against the ones obtained with CNNs that were specifically built for classifying the images of the PatchCamelyon dataset. The winner of the Kaggle competition obtained an AUC of 1, while the second-best performing network obtained an AUC of 0.98. On the other hand, the best performing network obtained with transfer learning (DenseNet architecture, with Adamax optimizer, and a learning rate of 10^−4^) was able to obtain an AUC of 0.95. This result confirms the suitability of transfer learning for the task at hand. More in detail, we believe that, by considering deeper architectures and more epochs, it could be possible to improve the results of this study, thus equaling the performance achieved by the winner of the Kaggle competition. On the other hand, we highlight a fundamental difference between the best performance reported in the present study and the best performance of the Kaggle competition: The former was obtained using an existing network (used for addressing different computer vision tasks) and by fine-tuning it, while the latter was achieved by designing an ad hoc CNN, a time-consuming task that requires some expertise.

## 6. Conclusions

CNN represents an analysis of images created using current computation techniques. This is mostly due to their ability to obtain a performance that is similar to, or better than, the one achieved by human beings. Nevertheless, similarly to other deep learning models, training a CNN is a task that usually requires a vast amount of images. This is an essential limitation in all the domains, like the medical one, in which data are scarce and difficult to obtain. In such a situation, transfer learning may provide a viable option. The idea of transfer learning is to use a model trained over thousands of observations (i.e., images in this study) to provide an initial architecture and set of weights for addressing a similar problem over a different domain. Motivated by the success of transfer learning in the analysis of medical images, and for further studying this promising research area, this paper compared the performance optimizers used in popular CNNs for the classification of histopathology images. In particular, four network architectures were used in the evaluation process. These networks were trained on the ImageNet dataset, which consists of millions of images, and their weights were fine-tuned to suit the considered histopathology images dataset. The results obtained from the experimental phase, in which different combinations of network, optimizer, and learning rate were considered, corroborated the initial hypothesis on the importance of the optimizer and the learning rate. While the choice of CNN is essential, it is clear that by fixing the value of the learning rate, the results obtained using different optimizers could be significantly different. On the other hand, once a particular optimizer is selected, the choice of the learning rate plays an essential role in determining the final performance of CNNs.

Interestingly, for each of the different CNNs under exam, it is possible to notice that the best performing configuration of optimizer and learning rate produces an AUC that is approximately 94%. This result strengthens the importance of selecting the hyperparameters of the network, and, in a future investigation, we will extend this work to include additional hyperparameters and datasets aiming at providing formal guidelines for medical experts that want to use CNN models to support their daily work.

## Figures and Tables

**Table 1:** Number of layers and parameters of the CNNs used in this study.

Networks	Number of Layers	Number of Parameters
VGG16	16	14,714,688
InceptionV3	48	21,802,784
ResNet50	50	23,587,712
DenseNet121	121	7,037,504

**Table 2:** Results obtained with the VGG16 architecture. Where LR stands for learning rate; NAG represents Nesterov momentum; AdaGrad represents the adaptive gradient optimizer; RMSProp represents the root mean square propagation optimizer; Adam represents adaptive moment estimation optimizer; AdaMax represents maximum adaptive moment estimation optimizer; and Nadam represents Nesterov and Adam optimizer.

Optimizers	$LR=10^{-3}$	$LR=10^{-4}$	$LR=10^{-5}$
NAG	89.45%	94.64%	94.25%
AdaGrad	88.50%	87.40%	88.07%
RMSProp	50.00%	94.33%	93.45%
Adam	90.88%	90.39%	95.01%
Adamax	50.00%	94.02%	94.20%
Nadam	85.00%	91.14%	94.33%

**Table 3:** Results obtained with the InceptionV3 architecture. Where LR stands for learning rate; NAG represents Nesterov momentum; AdaGrad represents the adaptive gradient optimizer; RMSProp represents the root mean square propagation optimizer; Adam represents adaptive moment estimation optimizer; AdaMax represents maximum adaptive moment estimation optimizer; and Nadam represents Nesterov and Adam optimizer.

Optimizer	$LR=10^{-3}$	$LR=10^{-4}$	$LR=10^{-5}$
NAG	93.18%	93.25%	90.81%
AdaGrad	93.64%	90.46%	86.32%
RMSProp	91.41%	94.91%	92.65%
Adam	90.44%	92.53%	93.22%
Adamax	93.44%	93.11%	93.95%
Nadam	91.97%	91.33%	92.46%

**Table 4:** Results obtained with the ResNet architecture. Where LR stands for learning rate; NAG represents Nesterov momentum; AdaGrad represents the adaptive gradient optimizer; RMSProp represents the root mean square propagation optimizer; Adam represents adaptive moment estimation optimizer; AdaMax represents maximum adaptive moment estimation optimizer; and Nadam represents Nesterov and Adam optimizer.

**Optimizer**	$LR=10^{-3}$	$LR=10^{-4}$	$LR=10^{-5}$
NAG	90.07%	93.84%	89.00%
AdaGrad	93.04%	89.11%	83.46%
RMSProp	89.56%	89.62%	93.04%
Adam	90.24%	90.24%	93.84%
Adamax	90.24%	92.24%	93.70%
Nadam	91.91%	89.36%	93.85%

**Table 5:** Results obtained with the DenseNet architecture. Where LR represents learning rate; NAG represents Nesterov momentum; AdaGrad represents the adaptive gradient optimizer; RMSProp represents the root mean square propagation optimizer; Adam represents adaptive moment estimation optimizer; AdaMax represents maximum adaptive moment estimation optimizer; and Nadam represents Nesterov and Adam optimizer.

**Optimizer**	$LR=10^{-3}$	$LR=10^{-4}$	$LR=10^{-5}$
NAG	93.08%	94.31%	91.64%
AdaGrad	93.89%	93.57%	87.70%
RMSProp	88.19%	93.98%	94.61%
Adam	84.18%	89.69%	94.43%
Adamax	90.62%	95.12%	93.91%
Nadam	86.77%	94.21%	93.77%
